# Blocking *Dirofilaria immitis* transmission by treatment of heartworm microfilaremic dogs with doxycycline

**DOI:** 10.1186/s13071-026-07383-4

**Published:** 2026-08-20

**Authors:** John Wilson McCall, Abdelmoneim Mansour, Utami DiCosty, Crystal Fricks, Scott McCall, Ben Carson, Michael Timothy Dzimianski, Charles Thomas Nelson, Andrew R. Moorhead

**Affiliations:** 1 TRS Labs, Inc., Athens, GA 30607 USA; 2 Department of Infectious Diseases, College of Veterinary Medicine, University of Georgia, Athens, GA 30602 USA; 3 Animal Medical Center, Anniston, AL 36268 USA; 4 Department of Clinical Sciences, College of Veterinary Medicine, North Carolina State University, Raleigh, NC 27607 USA

**Keywords:** *Dirofilaria immitis*, Mosquitoes, Migration, Development, Dogs, Doxycycline, Ivermectin, Blocking transmission

## Abstract

**Background:**

This study was conducted to determine whether heartworm infective larvae (L_3_) from mosquitoes fed earlier on blood from dogs during daily administration of doxycycline at various dosages and treatment schedules and a single prophylactic dose of ivermectin could develop normally in dogs.

**Methods:**

A total of 20 heartworm microfilaremic Beagles in a separate study allocated to four groups (*n* = 5/group) of five dogs were used as blood donors for mosquito infection. These donor dogs in groups 4, 5, and 6 had received doxycycline orally at 5.0, 7.5, or 10.0 mg/kg twice daily (BID) × 14 or 21 days, and ivermectin orally (minimum 6 mcg/kg) on day 0. Group 2 served as the untreated control. To produce infective larvae for each study, two sets of four groups of *Aedes aegypti* mosquitoes fed on blood from these four groups of dogs on day 14 (study A) or 21 (study B), respectively, and, infective third-stage larvae (L_3_) were recovered 14 days later. In each study, 8 heartworm-naive dogs (4 groups of 2) were inoculated subcutaneously with 50 L_3_/dog originating from mosquitoes fed on the corresponding donor group, for a total of 16 recipient dogs. Recipient dogs were monitored by antigen testing and microfilaria counts and necropsied for adult heartworm recovery and enumeration. Necropsies were performed on the same day at 252 days postinfection for study A and 245 days postinfection for study B.

**Results:**

Overall, 11 of the 12 dogs that had received L_3_ from mosquitoes fed on blood from treated dogs had no heartworms at necropsy; however, 1 dog that was administered doxycycline orally at the lowest dosage of 5.0 mg/kg BID daily and for the shorter treatment period of 14 days allowed only 1 adult female heartworm to reach the pulmonary arteries. All control dogs had male and female heartworms at necropsy.

**Conclusions:**

Treatment of microfilaremic dogs with doxycycline administered daily at 10.0 or 7.5 mg/kg BID for 14 days and one monthly dose of a heartworm preventive (macrocyclic lactone [ML]) renders the L_3_ incapable of normal development in recipient dogs, which could potentially widen the scope of a multimodal prevention approach to reduce the spread of heartworm disease.

**Graphical abstract:**

**Figure:**
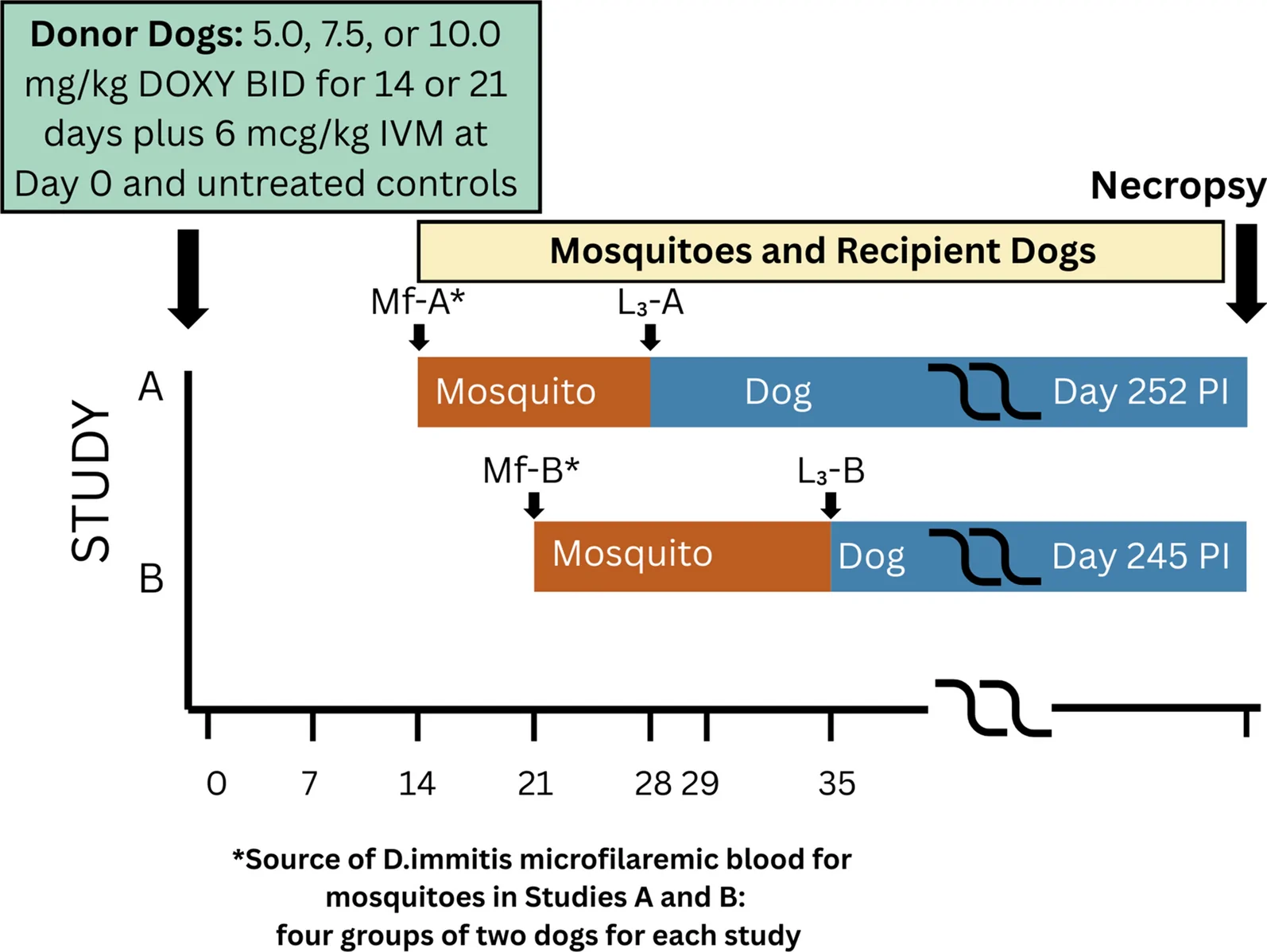

## Background

Some of the most important filarial parasites of animals and humans harbor *Wolbachia* endosymbionts (*Wolbachia* sp., Rickettsiaceae) [1–5], and every individual in every life cycle stage of these parasites has at least some of these bacteria [6, 7], even though they occur in varying proportions among individual worms and different developmental stages [8, 9]. These filariae and their endosymbiont share an obligatory mutualistic relationship in which the two organisms depend on each other for long-term well-being and eventually for survival [4, 10–14] and are important in immunity and disease [10, 15–22]. Several tetracycline drugs have deleterious effects on all life cycle stages, as well as the female reproductive system, of these filariae [4, 11, 16, 23–39], but have no adverse effects on filarial species, such as *Acanthocheilonema viteae*, that do not harbor *Wolbachia* [25, 27]. These drugs inhibit the development of the third-stage larvae (L_3_) and fourth-stage larvae (L_4_) [24, 25, 40]; are highly effective against immature adult worms [4, 36, 40]; have a slow killing effect against mature adult worms [14, 41–45]; lead to a block in embryogenesis [27, 28, 43, 46]; gradually eliminate microfilariae (Mf) from the circulating blood by attrition [11, 14, 47, 48]; and block transmission by rendering the surviving L_3_ collected from mosquitoes fed on blood from tetracycline-treated animals unable to later complete development in the final host [14, 43–45, 48–50]. Blocking of transmission by using tetracycline drugs has also been demonstrated to be due to killing the larval stages of *Litomosoides sigmodontis* in mites [49], *Brugia malayi* [51, 52] and *B. pahangi* [53] in mosquitoes, and *Onchocerca volvulus* in blackflies [54]. However, studies using sufficient dosage titrations and treatment period to investigate the killing of larval stages of *Dirofilaria immitis* in mosquitoes have not been reported [45].

Adding a macrocyclic lactone (ML) heartworm preventive drug to a doxycycline treatment protocol appears to have synergistic effects against *D. immitis* microfilariae and adult worms, as evidenced by more rapid elimination of circulating Mf and earlier killing of adult worms [14, 22, 39, 55–61]. However, the addition of ivermectin to the doxycycline protocol in our earlier *D. immitis* and *B. pahangi* studies in which we treated Mf-positive dogs did not appear to have enhanced killing effects on the Mf or L_3_, as L_3_ yields in the mosquitoes fed on blood from treated dogs seem to parallel the decreasing Mf counts in the blood donors, and efficacy in dogs inoculated subcutaneously (SC) with these L_3_ was 100% in all of our studies [14, 43, 45].

All of these earlier studies focused on the effects of doxycycline on the ability of L_3_ of *D. immitis* and *B. pahangi* collected from mosquitoes fed earlier on blood from treated and untreated microfilaremic dogs to survive and be inoculated SC into dogs. These studies examined blood collected during the last week (day 22 or 29) of a 1-month treatment regimen of doxycycline, with or without ivermectin, or at various times during the post-treatment microfilaremic period when Mf counts were declining. The objective of this study was to determine whether *D. immitis* L_3_ collected from mosquitoes fed on blood from microfilaremic dogs could develop normally and establish adult heartworm infections after daily administration of various dosages of doxycycline plus a single, prophylactic dose of ivermectin to microfilaremic dogs for only 14 days. A group treated with doxycycline for 21 days and given a single dose of ivermectin was included to confirm data on a previous study [50].

## Methods

### General study design

#### Infection and treatment of microfilaremic donor dogs

The 20 adult Beagle dogs, selected from a total of 45 dogs from a different study [62], that were used as blood donors in the current study were each infected with *D. immitis* (Georgia III isolate) by intravenous (IV) transplantation via a jugular vein of adult worms from donor dogs. The adult worms were transplanted within a few hours after recovery from the donor dogs, with replacements of fresh Hanks’ balanced salt solution as needed to maintain the proper pH prior to transplantation of the worms [63]. Subsequently, 75 days later, they were randomly allocated to four groups of five dogs each. Starting on day 0, groups 4, 5, and 6 were administered doxycycline orally at 5.0, 7.5, and 10.0 mg/kg twice daily (BID) for 28 days, respectively, and given a dose of ivermectin (minimum 6 mcg/kg) on study days 0 and 28. Please note that the doses of doxycycline administered on days 22–28 and the dose of ivermectin given on day 30 were irrelevant here because blood for the current study was drawn before day 28. Group 2 served as the untreated control. Please note that the group numbers for the treated-donor-dog study do not correspond to the group numbers for the current study. The 15 dogs in the treated groups and 5 dogs in the untreated group served as the microfilaremic blood source for the mosquitoes used in the current study (study A and study B). For further details, such as age, weight, sex, source, animal management, etc. regarding these dogs, see data published elsewhere [62].

#### Subcutaneous inoculation of heartworm-naïve dogs with L_3_ collected from mosquitoes, antigen tests, microfilaria tests, and necropsy of dogs

A total of 16 uninfected recipient Beagle dogs for the current study were randomly allocated to two sets (A and B) of four groups of two dogs each, respectively. Separate batches of mosquitoes, corresponding to the appropriate test and control groups, were labeled for feeding on blood from donor dogs treated with ivermectin on day 0 and doxycycline at 10.0 (test groups A-1, B-1), 7.5 (test groups A-2, B-2), and 5.0 (test groups A-3, B3) mg/kg BID for 14 or 21 days and one untreated group per study (test groups A-4, B-4). Thus, four sets of *Aedes aegypti* mosquitoes were allowed to feed on group-pooled blood samples from donor dogs for three test groups (A-1, A-2, and A-3) and one control group (A-4) on day 14 (study A, eight dogs). Another four sets of mosquitoes were allowed to feed on blood from the same donor dogs on day 21 (study B, eight dogs) for three test groups (B-1, B-2, and B-3) and one control group (B-4). The number of microfilariae in the pooled blood samples for each membrane feeder was approximately 50 Mf/20 mcL. If the count was 100 Mf/20 mcL or more, blood without microfilariae was added to reduce the count to approximately 50 Mf/20 mcL. The reason for doing this is that the microfilariae will kill the mosquitoes if the count is too high. Then, 14 days after each feeding (day 28, study A; day 35, study B), the 8 recipient dogs in each study (16 total recipient dogs) were inoculated SC with 50 L_3_ from the appropriate batch of mosquitoes (Tables 1 and 2). Blood was collected from all recipient dogs for performing microfilaria counts and antigen tests prior to inoculation, at 6 months postinoculation (PI) and every other week thereafter until necropsy. Although study A started 1 week earlier than study B, all 16 recipient dogs were euthanized on the same day, and a necropsy was performed for recovery and enumeration of adult heartworms, i.e., at 252 days PI (study A) and 245 days PI (study B). Length measurement was carried out on 5 female worms chosen randomly from each of the 4 control dogs (total of 20 worms) and a total of 1 surviving worm from the total of 12 dogs in the six test groups.

**Table 1:** *D. immitis* Mf counts, Ag scores, and worm counts for dogs inoculated with L_3_ from mosquitoes fed on blood from doxycycline-treated dogs

				Mf counts per mL/Ag scores at months PI^a^						No. of adult heartworms	
Group	Dog^b^	Mosq. fed on day	Doxy^c^	0	6	6.5	7	7.5	8	Nec.^d^ day	Adult worms
A-1	1, 2	14	10	0/Neg	0/Neg	0/Neg	0/Neg	0/Neg	0/Neg	252	0, 0
A-2	3, 4	14	7.5	0/Neg	0/Neg	0/Neg	0/Neg	0/Neg	0/Neg	252	0, 0
A-3	5^e^, 6	14	5	0/Neg	0/Neg	0/Neg	0/Neg	0/Neg	0/Neg	252	1, 0
B-1	9, 10	21	10	0/Neg	0/Neg	0/Neg	0/Neg	0/Neg	0/Neg	245	0, 0
B-2	11, 12	21	7.5	0/Neg	0/Neg	0/Neg	0/Neg	0/Neg	0/Neg	245	0, 0
B-3	13, 14	21	5	0/Neg	0/Neg	0/Neg	0/Neg	0/Neg	0/Neg	245	0, 0

^a^All dogs had no microfilariae per mL of blood (0) and were negative (Neg) on the antigen test on all days tested

^b^50 L_3_/dog

^c^Dosages in mg/kg BID administered orally for 14 or 21 days

^d^Nec. day = necropsy day

^e^Small female worm. Mosq., mosquitoes; doxy, doxycycline

**Table 2:** *D. immitis* Mf counts, Ag scores, and worm counts for dogs infected with L_3_ from mosquitoes fed on blood from untreated dogs

			Mf counts/Ag scores at months PI^a^					No. of adult heartworms			
Group	Dog^b^	Mosq. fed on day	6^c^^,e^	6.5^d,e^	7^d^^,e^	7.5^d,e^	8^d^^,e^	Nec. day^f^	Male	Female	Total
A-4	7	14	42/Pos	48/Pos	62/Pos	179/Pos	215/Pos	252	16	19	35
A-4	8	14	71/Pos	22/Pos	95/Pos	140/Pos	152/Pos	252	13	15	28
B-4	15	21	4/Pos	6/Pos	65/Pos	131/Pos	179/Pos	245	20	23	43
B-4	16	21	20/Pos	13/Pos	76/Pos	218/Pos	247/Pos	245	8	8	16

^a^All dogs had no microfilariae per mL of blood and were negative on the antigen test at day 0

^b^50 L^3^/dog

^c^Mf counts per mL of blood

^d^Mf counts per 20 mcL of blood (too many Mf to count in 1.0 mL of blood)

^e^Pos = positive on the heartworm antigen test

^f^Nec. day = necropsy day

#### Mosquito strain and heartworm isolate

The black-eyed Liverpool strain of *Aedes aegypti* used in this study was obtained from Prof. W.W. Macdonald at the Department of Parasitology and Entomology at the Liverpool School of Tropical Medicine (England) in 1972 by Dr. John W. McCall and the late Dr. Paul E. Thomson at the University of Georgia (UGA). Dr. Macdonald had obtained the strain from West Africa in 1961. TRS Labs, Inc. (TRS) obtained the strain from UGA in 1980. It was estimated that this strain has been maintained through 2002 generations since it was established in the UGA laboratory in 1972. For additional information on this strain, see [44].

The Georgia III isolate of *D. immitis* was used in this study. This isolate was obtained by TRS from Oconee County, Georgia, USA on 9 October 2017 and was validated by testing positive for heartworm microfilariae and antigen, and by worms recovered at necropsy in June of 2018. It is known to be susceptible to ML heartworm preventives [64].

#### Animals and animal management

In total, 16 purpose-bred male (8) and female (8) Beagle dogs from a commercial supplier were used in this study (study A and study B). They ranged in age from 4.8 to 15.0 months and ranged in body weight from 5.9 to 11.7 kg on the day of infection of the first set of 8 dogs, i.e., study A. They were born and raised indoors in mosquito-proof facilities and were not treated with any heartworm-preventive drugs prior to the start of this study. They had negative test results for microfilariae (modified Knott test) and adult heartworm antigen (DiroCHEK™ Heartworm Antigen Test Kit, Zoetis, Inc., Kalamazoo, MI, USA) and were randomly allocated by a table of random numbers to eight groups of two dogs each just prior to inoculation of the first set of dogs. The dogs were housed individually in 4 ft. × 5 ft. kennels or pair-housed by gender within groups with access to two kennels. The dogs received at least one daily health observation each day throughout the study. All animals were maintained with due regard for their welfare. This study was approved under AUP 22-04 and AUP 23-04 by the TRS Institutional Animal Care and Use Committee prior to initiation of the study.

#### Study drugs

Doxycycline hyclate was administered orally to the treated microfilaremic donor dogs (i.e., those in the related parent study) as 20, 50, and 100 mg tablet (Epic Pharma, Avec Pharma) to provide dosages of approximately 10.0, 7.5, or 5.0 mg/kg twice daily. The availability of 20 mg tablets allowed for more accurate dosing at the target dosage.

Ivermectin was administered orally as 68 mcg chewables (Heartgard^®^, Boehringer Ingelheim, Duluth, GA, USA) to the same dogs on day 0 (of the related parent study) to achieve a minimum dosage of 6.0 mcg/kg of ivermectin. The average dose of ivermectin administered on day 0 was 7.57 mcg/kg, with a range of 6.02–9.10 mcg/kg. Any dosing of the treated donor dogs in the related parent study after 21 days of dosing was irrelevant, as the “donated” microfilaremic blood used in the present study was collected on days 14 and 21 after dosing started. No recipient dogs in the present transmission study were treated.

#### Statistical analysis

A statistical analysis of the data was not performed. Only 1 of the 12 dogs in the six treated test groups (A-1, A-2, A-3, B-1, B-2, and B-3) had any live or dead worms at necropsy and this dog (5), which was in A-3, had only one small, adult female heartworm. In contrast, all four dogs in both untreated control groups (A-4 and B-4) had heartworms.

## Results

### Recovery of adult heartworms at necropsy

All three dosages of doxycycline used in both treatment schedules were highly effective (Table 1). All dogs in test groups B-1, B-2, and B-3 that received doxycycline treatment at 10.0, 7.5, and 5.0 mg/kg, respectively, for 21 days had no worms at necropsy; thus, the drug was 100% effective in all of these test groups by day 21. All dogs in test groups A-1 and A-2 that received doxycycline treatment at 10.0 and 7.5 mg/kg, respectively, for 14 days had no worms at necropsy; thus, the drug was 100% effective in these two test groups. The 5.0 mg/kg doxycycline treatment (A-3) for 14 days was highly effective: one dog that received the doxycycline treatment in test group A-3 had no worms, but one dog had one worm. This female worm in test group A-3 measured 22.0 cm in length, while the average length of 20 females (5 worms from each of the 4 dogs) in untreated control groups A-4 and B-4 was 26.1 cm, with a range of 21.4–29.5 cm (measurement data not included in the tables). No worms were recovered from any of the remaining 11 dogs in the six treated test groups. The 4 untreated control dogs had a mean of 30.5 adult male and female worms per dog, with a range of 16–43 (Table 2).

### Heartworm antigen and microfilaria test results

None of the 12 dogs in the six test groups had microfilariae or were positive for adult heartworm Ag during the study (Table 1), while all 4 dogs in the untreated control groups were positive for Mf and Ag at all the postinfection days tested (Table 2).

## Discussion

As mentioned earlier, treatment of animals and humans with tetracycline drugs, particularly doxycycline, has profound adverse effects on all life cycle stages of filariae that harbor *Wolbachia.* Doxycycline inhibits the development of L_3_ and L_4_; is highly effective against immature adult worms; has a slow killing effect on mature adult worms; and disrupts embryogenesis and gradually eliminates Mf from the circulating blood or skin by attrition. It is informative to note that daily administration of doxycycline at 10 mg/kg once daily (SID) for 30 days plus a single prophylactic dose of ivermectin (minimum 6 mcg/kg) on day 0 to *D. immitis* microfilaremic dogs prohibited a rebound in Mf count after day 239 of a 647-day study [44]. Treatment of microfilaremic animals with tetracyclines also inhibits or reduces the development of Mf to the L_3_ stage in mosquitoes and renders the Mf collected from these mosquitoes unable to complete normal development when L_3_ from the mosquitoes are inoculated into dogs. These Mf and L_3_ are normal in appearance and motility, but the L_3_ cannot develop and migrate to their preferred location in the definitive hosts.

In all of our earlier studies with *D. immitis*, blood collected from microfilaremic dogs starting as early as 22 days after beginning treatment with doxycycline at 10 mg/kg SID in one study [50] and continuing periodically until the Mf level approached zero in other studies [14, 43] showed that the L_3_ collected from these mosquitoes and inoculated SC into dogs were incapable of developing and migrating to the heart and lung arteries.

Similar results were obtained with *B. pahangi* in dogs [45]. No worms were recovered from the lymphatics after L_3_ collected from mosquitoes fed on blood from dogs treated with doxycycline were inoculated into dogs. However, because *B. pahangi* will develop in the peritoneal cavity of Mongolian jirds [65], we were able to obtain valuable information on the development and length of survival of L_3_ collected from these mosquitoes and inoculated IP into jirds [45]. Compared with untreated controls, 19.0% and 1.7% of the immature larvae and young adults of *B. pahangi* recovered from jirds necropsied at 35 and 60 days PI, respectively, survived, and stunting of their growth was noted. In all of our similar studies with *D. immitis* [14, 43, 50], we have not attempted to do such a study in dogs owing to the difficulties in finding very small migrating worms in the body tissues. In our *B. pahangi* studies, we were able to circumvent this problem by using intraperitoneal infections in jirds, where most of the worms of all stages are confined to the readily accessible abdominal cavity. This jird model allows investigators to reduce the number of dogs, cats, and ferrets needed for laboratory research on lymphatic-dwelling *B. pahangi* and *B. malayi*. Fortunately, a *D. immitis* rodent model that could be used for selected research projects is currently under development and shows much promise [64].

The blocking-of-transmission effect is dose and treatment-length dependent. In the current study, transmission of *D. immitis* was completely blocked by oral treatment of microfilaremic blood donor dogs with doxycycline administered at 10.0, 7.5, and 5.0 mg/kg BID for 21 days and at both 10 mg/kg BID and 7.5 mg/kg BID for 14 days, but one small female worm was recovered from one dog in test group A-3 that was administered the drug at a lower dosage of 5 mg/kg BID for 14 days. Whether this inhibition of transmission would occur following administration of 10 mg/kg BID or 7.5 mg/kg BID for less than 14 days is not known.

*Wolbachia* are reported to be an essential source of nucleotides and micronutrient pathways (FAD, heme, and riboflavin) to support the high demand on biosynthesis during rapid oogenic/embryonic cell division [12] in the germline *Wolbachia* population. Depletion of the *Wolbachia* may lead to deficiency in growth and survival factors needed for embryogenesis and induces apoptosis of embryonic and uterine tissues, which would result in sterility [66].

*B. malayi* Mf depleted of *Wolbachia* will not develop in the mosquito owing to a defect in exsheathment of the Mf, which prohibits migration through the gut wall of the mosquito. Although this elimination of *Wolbachia* reduces the ability of the Mf to infect the mosquito, they remain motile and alive [38, 49, 66]. There is also a dose-dependent reduction in *Wolbachia* levels with 2-, 4-, and 6-week courses of tetracycline treatment that correlates with the level of transmission blocking. The low dose of 2 mg/kg daily for 2 weeks reduces the *Wolbachia* load in Mf by 35% and development of L_3_ in the mosquito by 67%, while the 6-week course of treatment results in a 79–100% reduction of L_3_ recovery rate following 90% removal of *Wolbachia* [51].

To study this block in transmission in the mosquito, Quek et al. [52] identified a relatively small number of genes in *B. malayi* microfilariae that showed differential expression. Genes related to chitinase activity were identified as part of a set of downregulated genes. Some filarial species have chitinase enzymes that are critical for development [66]. See Quek et al. [52] for a discussion on the critical role of chitinase on the exsheathment of Mf in some species, e.g., *B. malayi* [67–69], that have a sheath and L_3_ in some species that are unsheathed, e.g., *D. immitis* [70] and *O. volvulus* [71].

Anti-*Wolbachia* drugs should be reconsidered as having a macrofilaricide + microfilaricide pharmacological profile and not as a macrofilaricide only. The reason for this is that *Wolbachia*-depleted Mf results in a 79–100% block in transmission. Thus, *Wolbachia* elimination from Mf can exert both short-term and long-term suppression of transmission, while avoiding the side effects of microfilaricide treatment [19, 20, 51, 71, 72].

The inhibition of transmission in some filarial species that harbor *Wolbachia* by antibiotic treatment is due to both killing of larvae in the vector as well as rendering L_3_ from the vector incapable of developing in the vertebrate host. In our studies on *D. immitis* [14, 43, 50] and *B. pahangi* [45], we did not quantify or analyze mosquito infection intensity and/or L_3_ yields. However, even following administration of doxycycline at a dosage as high as 10 mg/kg twice daily for 28–30 days and feeding mosquitoes blood collected during treatment as well as at numerous times during the decline in Mf counts after treatment, our empirical observations suggest that lower recovery of L_3_ in these experiments is due to fewer Mf in the blood imbibed by the mosquito, rather than to the death of larvae in the mosquitoes. This suggests that antibiotic treatment at the dosages and treatment schedules used in the current study may not lead to killing of microfilariae and/or maturing larvae in the mosquito and L_3_ in the vertebrate host in every species of filariae that harbor *Wolbachia* [45].

There are several limitations to the interpretation of the data in this study. First, a small number of recipient dogs in the six test groups (*n* = 2) reduces the level of confidence in the 100% reduction in worm burden in 11 of the 12 dogs in five of the test groups and near 100% reduction in worm burden in the remaining test group in the present study. The large number of test dogs with 100% reduction in worm burden in other similar studies [14, 43, 45, 50) and the lack of any breakthroughs strongly supports a robust claim. However, a follow-up study with a statistically valid number of dogs per group is needed to increase the level of confidence in 14 and 21 days of treatment with doxycycline plus a single dose of ivermectin in the present study. Second, only one isolate of *D. immitis* (GA III) was used in the present study. However, several other isolates (Butch, Lady, Missouri [14]; MP 3, Michigan, Pepper [43]; and Berkeley [50]) have been used with 100% reduction of worm burden in other heartworm studies. Third, only the long-term laboratory strain of *Aedes Aegypti* (blackeyed Liverpool) was used in the present study, and this may not represent all laboratory strains and species of mosquitoes. This study should be repeated using other laboratory-maintained species of mosquitoes, such as *Aedes albopictus*, *Aedes togoi*, *Anopheles quadrimaculatus*, or *Culex fatigans*. Fourth, data on experimental subcutaneous inoculation of recovered L_3_ instead of natural transmission to recipient dogs may not extrapolate to field conditions. For example, to more closely simulate natural conditions, selected parts of studies similar to the current study could be conducted by feeding mosquitoes directly on treated microfilaremic dogs in a laboratory or field study and then allowing these L_3_-infected mosquitoes to feed on recipient dogs in the laboratory or under field conditions.

The American Heartworm Society recommends the inclusion of doxycycline along with a macrocyclic lactone treatment prior to administration of melarsomine, in a heartworm adulticide treatment protocol [73]. This should reduce pathology associated with dead worms [59–61], kill most larval and adult stages [40], and disrupt heartworm transmission [14, 43, 50]. The inclusion of doxycycline or another antibiotic that is active against *Wolbachia* sp. in filariae in an antifilarial treatment protocol, as part of a multimodal risk-management program, would have numerous beneficial effects. It would not only reduce the pathology and the larva, immature, and mature adult worm burdens, and gradually eliminate Mf in the mammalian host, but also contribute substantially to reduction in the spread of the most important filarial diseases in dogs (e.g., *D. immitis*) and humans (e.g., *B. malayi*, *B. timori*, *Wucherieria bancrofti*, *O. volvulus*) in parts of the world where they are endemic by blocking transmission [45].

## Conclusions

Data from this study support the inclusion of doxycycline and an ML in the American Heartworm Society’s recommended heartworm adulticide protocol. It adds the significant epidemiological benefit of blocking the normal development of heartworm L_3_, even ML-resistant biotypes, transmitted to the animal host.

## Data Availability

The data included in this manuscript were generated by TRS Laboratories, Inc. Data supporting the main conclusions of this study are included in the manuscript.

## Abbreviations

BID: Administered twice daily
SID: Administered once daily
L_3_: Third-stage larvae
L_4_: Fourth-stage larvae
IV: Intravenous
IP: Intraperitoneally
Mf: Microfilariae
ML: Macrocyclic lactone
SC: Subcutaneously
PI: Postinoculation
AG: Antigen
AUP: Animal use proposal

## References

[1] Sironi M, Bandi C, Sacchi L, Di Sacco B, Damiani G, Genchi C. A close relative of the arthropods endosymbiont *Wolbachia* in a filarial worm. Mol Biochem Parasitol. 1995;74:223–7. 8719164 10.1016/0166-6851(95)02494-8

[2] Casiraghi M, Anderson TJC, Bandi C, Bazzocchi C, Genchi C. A phylogenetic analysis of filarial nematodes: comparison with the phylogeny of *Wolbachia* endosymbionts. Parasitology. 2001;122:93–103. 11197770 10.1017/s0031182000007149

[3] Casiraghi M, Bain O, Guerrero R, Martin C, Pocacqua V, Gardner SL, et al. Mapping the presence of *Wolbachia pipientis* on the phylogeny of filarial nematodes: evidence for symbiont loss during evolution. Int J Parasitol. 2004;34:191–203. 15037105 10.1016/j.ijpara.2003.10.004

[4] Taylor MJ, Bandi C, Hoerauf A. *Wolbachia* bacterial endosymbionts of filarial nematodes. Adv Parasitol. 2005;60:248–86. 10.1016/S0065-308X(05)60004-8 16230105

[5] Kramer LH, Passeri B, Corona S, Simoncini L, Casiraghi M. Immunohistochemical/immunogold detection and distribution of the endosymbiont *Wolbachia* of *D. immitis* and *Brugia pahangi* using a polyclonal antiserum raised against WSP (*Wolbachia* surface protein). Parasitol Res. 2003;89:381–6. 12632152 10.1007/s00436-002-0765-6

[6] Kozek WJ. Transovarially-transmitted intracellular microorganisms in adult and larval stages of *Brugia malayi*. J Parasitol. 1977;63:992–1000. 592054

[7] Kozek WJ, Figueroa MH. Intracytoplasmic bacteria in *Onchocerca volvulus*. Am J Trop Med Hyg. 1977;26:663–78. 889009 10.4269/ajtmh.1977.26.663

[8] McGarry HF, Egerton G, Taylor MJ. Population dynamics of *Wolbachia* bacterial endosymbionts in *Brugia malayi*. Mol Biochem Parasitol. 2004;135:57–67. 15287587 10.1016/j.molbiopara.2004.01.006

[9] Fenn K, Blaxter M. Quantification of *Wolbachia* in *Brugai malayi* through the nematode lifecycle. Mol Biochem Parasitol. 2004;137:361–4. 15383308 10.1016/j.molbiopara.2004.06.012

[10] Taylor MJ, Cross HF, Ford L, Makunde WJ, Prasad GBKS, Bilo K. *Wolbachia* bacteria in filarial immunity and disease. Par Immunol. 2001;23:401–9. 10.1046/j.1365-3024.2001.00400.x 11472559

[11] Taylor MJ, Makunde WH, McGarry HF, Turner JD, Mand S, Hoerauf A. Macrofilaricidal activity after doxycycline treatment of *Wuchereria bancrofti*: a double-blind, randomised placebo-controlled trial. Lancet. 2005;365:2116–21. 15964448 10.1016/S0140-6736(05)66591-9

[12] Taylor MJ, Veronin D, Johnston KL, Ford L. *Wolbachia* filarial interactions. Cell Microbiol. 2013;15:520–6. 23210448 10.1111/cmi.12084

[13] Hise AG, Gillette-Ferguson I, Pearlman E. The role of endosymbiotic *Wolbachia* bacteria in filarial disease. Cell Microbiol. 2004;6:97–104. 14706096 10.1046/j.1462-5822.2003.00350.x

[14] McCall JW, Genchi C, Kramer L, Guerrero J, Dzimianski MD, Supakorndej P, et al. Heartworm and *Wolbachia:* therapeutic implications. Vet Parasitol. 2008;158:204–14. 18930598 10.1016/j.vetpar.2008.09.008

[15] Bazzocchi C, Ceciliani F, McCall JW, Ricci I, Genchi C, Bandi C. Antigenic role of the endosymbionts of filarial nematodes: IgG response against the *Wolbachia* surface protein in cats infected with *Dirofilaria immitis*. Proc R Soc London B. 2000;267:1–6. 10.1098/rspb.2000.1313 PMC1690852 11197127

[16] Genchi C, Simoncini L, McCall JW, Venco L, Lo N, Bazzocchi C, et al. The *Wolbachia* endosymbionts of *Dirofilaria immitis*: implications for pathology, immunology and control. In: Seward RL, editor., et al., Recent advances in heartworm disease: symposium ’01. Batavia, Illinios: American Heartworm Society; 2001. p. 27–33.

[17] Brattig NW, Buttner DW, Hoerauf A. Neutrophil accumulation around *Onchocerca* worms and chemotaxis of neutrophils are dependent on *Wolbachia* endobacteria. Microbes Infect. 2001;3:439–46. 11377205 10.1016/s1286-4579(01)01399-5

[18] Brattig NW, Bazzocchi C, Kirschning CJ, Reiling N, Bϋttner DW, et al. The major surface protein of *Wolbachia* endosymbionts in filarial nematodes elicits immune responses through TLR2 and TLR4. J Immunol. 2004;73:437–45. 10.4049/jimmunol.173.1.437 15210803

[19] Turner JD, Langley RS, Johnston KL, Egerton G, Wanji S, Taylor MJ. *Wolbachia* endosymbiotic bacteria of *Brugia malayi* mediated macrophage tolerance to TLR- and CD40-specific stimuli in a MyD88/TLR2-dependent manner. J Immunol. 2006;174:1240–9. 10.4049/jimmunol.177.2.1240 16818783

[20] Turner JD, Mand S, Debrah AY, Muehlfeld J, Pfarr K, McGarry HF, et al. A randomized, double-blind clinical trial of a 3-week course of doxycycline plus albendazole and ivermectin for the treatment of *Wuchereria bancrofti* infection. Clin Infect Dis. 2006;42:1081–9. 16575724 10.1086/501351

[21] Grandi G, Kramer LH, Bazzocchi C, Mortarino M, Venco L, Genchi C. *Dirofilaria immitis* and the endosymbiont *Wolbachia*: inflammation and immune response in heartworm disease. Jap J Vet Parasitol. 2005;4:11–6.

[22] Kramer LH, Grandi G, Leoni M, McCall J, Bazzochi M, Mortarino, et al. *Wolbachia* and its influence on the pathology and immunology of *Dirofilaria immitis* infection. Vet Parasitol. 2008;158:191–5. 18947926 10.1016/j.vetpar.2008.09.014

[23] Bandi C, McCall JW, Genchi C, Corona S, Venco L, Sacchi L. Effects of tetracycline on the filarial worms *Brugia pahangi* and *Dirofilaria immitis* and their bacterial endosymbiont *Wolbachia*. Int J Parasitol. 1999;29:357–64. 10221636 10.1016/s0020-7519(98)00200-8

[24] Bosshardt SC, McCall JW, Coleman SU, Jones KL, Petit TA, Klei TR. Prophylactic activity of tetracycline against *Brugia pahangi* in jirds (*Meriones unguiculatus*). J Parasitol. 1993;79:775–7. 8410553

[25] McCall JW, Jun JJ, Bandi C. *Wolbachia* and the antifilarial properties of tetracycline. An untold story. Ital J Zool. 1999;66:7–10.

[26] Townson S, Hutton D, Siemienska J, Hollick L, Scanlon T, Tagoboto SK, et al. Antibiotics and *Wolbachia* in filarial nematodes: antifilarial activity of rifampicin, oxytetracycline and chloramphenicol against *Onchocerca gutturosa*, *Onchocerca lienalis* and *Brugia pahangi*. Ann Trop Med Parasitol. 2000;94:801–16. 11214099 10.1080/00034980020027988

[27] Hoerauf A, Nissen-Pahle K, Schmetz C, Henkle-Duhrsen K, Blaxter ML, Bϋttner DW, et al. Tetracycline therapy targets intracellular bacteria in the filarial nematode *Litomsoides sigmodontis* and results in filarial infertility. J Clin Invest. 1999;103:11–8. 9884329 10.1172/JCI4768 PMC407866

[28] Hoerauf A, Volkmann L, Hamelmann C, Adjei O, Autenrieth IB, Fleischer B, et al. Endosymbiotic bacteria in worms as targets for a novel chemotherapy in filariasis. Lancet. 2000;355:1242–3. 10770311 10.1016/S0140-6736(00)02095-X

[29] Hoerauf A, Volkmann L, Nissen-Paehle K, Schmetz C, Autenrieth I, Bϋttner DW, et al. Targeting of *Wolbachia* endobacteria in *Litomosoides sigmodontis*: comparison of tetracyclines with chloramphenicol, macrolides and ciprofloxacin. Trop Med Internat Hlth. 2000;5:275–9. 10810023

[30] Hoerauf A, Mand S, Adjei O, Fleischer B, Bϋttner DW. Depletion of *Wolbachia* endobacteria in *Onchocerca volvulus* by doxycycline and microfilaridermia after ivermectin treatment. Lancet. 2001;357:1415–6. 11356444 10.1016/S0140-6736(00)04581-5

[31] Hoerauf A, Mand S, Volkmann L, Bϋttner M, Marfo-Debrekyei Y, Taylor M, et al. Doxycycline in the treatment of human onchocerciasis: kinetics of *Wolbachia* endobacteria reduction and inhibition of embryogenesis in female *Onchocerca* worms. Microb Infect. 2003;5:261–73. 10.1016/s1286-4579(03)00026-1 12706439

[32] Hoerauf A, Mand S, Fischer K, Kruppa T, Marfo-Debrekyei Y, Debrah A, et al. Doxycycline as a novel strategy against bancroftian filariasis-depletion of *Wolbachia* endosymbionts from *Wuchereria bancrofti* and stop of microfilaria production. Med Microbiol Immunol. 2003;192:211–6. 12684759 10.1007/s00430-002-0174-6

[33] Casiraghi M, McCall JW, Simoncini L, Kramer LH, Sacchi L, Genchi C, et al. Tetracycline treatment and sex-ratio distortion; a role for *Wolbachia* in the moulting of filarial nematodes? Int J Parasitol. 2002;32:1457–68. 12392911 10.1016/s0020-7519(02)00158-3

[34] Chirgwin SR, Nowling JM, Coleman SU, Klei TR. *Brugia pahangi* and *Wolbachia*: the kinetics of bacteria elimination, worm viability, and host responses following tetracycline treatment. Exp Parasitol. 2003;103:16–26. 12810042 10.1016/s0014-4894(03)00063-8

[35] Kozek WJ. What is new in the *Wolbachia/Dirofilaria* interactions? Vet Parasitol. 2005;133:127–32. 16198819 10.1016/j.vetpar.2005.02.005

[36] Langworthy N, Renz A, Meckenstedr U, Henkle-Duhrsen K, de Bronvoort M, Tanya V, et al. Macrofilaricidal activity of tetracycline against the filarial nematode *Onchocerca ochengi*: elimination of *Wolbachia* precedes worm death and suggests a dependent relationship. Proc R Soc London Series B Bio Sci. 2000;267:1063–9. 10.1098/rspb.2000.1110 PMC1690645 10885510

[37] Volkmann L, Fischer K, Taylor M, Hoerauf A. Antibiotic therapy in murine filariasis (*Litomosoides sigmodontis*): comparative effects of doxycycline and rifampicin on *Wolbachia* and filarial viability. Trop Med Int Health. 2003;8:392–401. 12753632 10.1046/j.1365-3156.2003.01040.x

[38] Debrah AY, Mand S, Marfo-Debrekyei Y, Batsa L, Pfarr K, Buttner M, et al. Macrofilaricidal effect of 4 weeks of treatment with doxycycline on *Wuchereria bancrofti*. Trop Med Int Health. 2007;12:1433–41. 18076549 10.1111/j.1365-3156.2007.01949.x

[39] Grandi C, Quintavalla C, Mavropoulou A, Genchi M, Gnudi G, Bertoni G, et al. A combination of doxycycline and ivermectin is adulticidal in dogs with naturally acquired heartworm disease (*Dirofilaria immitis*). Vet Parasitol. 2010;169:347–51. 20144506 10.1016/j.vetpar.2010.01.025

[40] McCall JW, Kramer LH, Genchi C, Guerrero J, Dzimianski MT, Supakorndej P, et al. Effects of doxycycline on early infections of *Dirofilaria immitis* in dogs. Vet Parasitol. 2011;176:361–7. 21345592 10.1016/j.vetpar.2011.01.022

[41] McCall JW, Genchi C, Kramer LH, Guerrero J, Venco L. Heartworm disease in animals and humans. Adv Parasitol. 2008;66:193–285. 18486691 10.1016/S0065-308X(08)00204-2

[42] Gilbert J, Nfon CK, Makepeace BL, Njongmeta LM, Hastings IM, Pfarr KM, et al. Antibiotic chemotherapy of onchocerciasis: in a bovine model, killing of adult parasites requires a sustained depletion of endosymbiotic bacteria (*Wolbachia* species). J Infect Dis. 2005;192:1483–93. 16170768 10.1086/462426

[43] McCall JW, Kramer L, Genchi C, Guerrero J, Dzimianski MT, Mansour A, et al. Effects of doxycycline on heartworm embryogenesis, transmission, circulating microfilaria, and adult worms in microfilaremic dogs. Vet Parasitol. 2014;206:5–13. 25458121 10.1016/j.vetpar.2014.09.023

[44] McCall JW, DiCosty U, Mansour A, Fricks C, McCall S, Dzimianski M, et al. Inability of *Dirofilaria immitis* infective larvae from mosquitoes fed on blood from microfilaremic dogs during low-dose and short-treatment regimens of doxycycline and ivermectin to complete normal development in heartworm naive dogs. Parasit Vectors. 2023;16:199. 37312202 10.1186/s13071-023-05704-5 PMC10262448

[45] McCall J, Kramer L, Genchi C, Guerrero J, Dzimianski MT, Mansour A, et al. Effects of doxycycline on prepatent and patent infections of *Brugia pahangi* in dogs and observations on the growth and survival of L_3_ in jirds and dogs. Vet Parisitol. 2025. 10.1016/j.vetpar.2025.110568. 10.1016/j.vetpar.2025.110568 40782506

[46] Genchi C, Sacchi L, Bandi C, Venco L. Preliminary results on the effect of tetracycline on the embryogenesis and symbiotic bacteria (*Wolbachia*) of *Dirofilaria immitis.* An update and discussion. Parasitologia. 1998;40:247–9. 10376278

[47] Slatko BE, Taylor MJ, Foster JM. The *Wolbachia* endosymbiont as an anti-filarial nematode target. Symbiosis. 2010;51:55–65. 20730111 10.1007/s13199-010-0067-1 PMC2918796

[48] Supali T, et al. Doxycycline treatment of *Brugia malayi*-infected persons reduces microfilaremia and adverse reactions after diethylcarbamazine and albendazole treatment. Clin Infect Dis. 2008;46:1385–93. 18419441 10.1086/586753

[49] Arumugam S, Pfarr KM, Hoerauf A. Infection of the intermediate host with *Wolbachia*-depleted *Litomosoides sigmodontis* microfilariae: impaired L_1_ to L_3_ development and subsequent sex-ratio distortion in adult worms. Int J Parasitol. 2008;38:981–7. 18282572 10.1016/j.ijpara.2007.12.006

[50] McCall JW, Mansour A, DiCosty U, Fricks C, McCall S, Dzimianski MT, et al. Long-term evaluation of viability of microfilariae and IV transplanted adult *Dirofilaria immitis* in microfilaremic dogs treated with low-dose, short- and long-treatment regimens of doxycycline and ivermectin. Parasites Vectors. 2023;16:190. 37291586 10.1186/s13071-023-05769-2 PMC10251710

[51] Srivastava K, Misra-Bhattacharya S. Tetracycline, a tool for transmission blocking of *Brugia malayi* in *Mastomys coucha*. Curr Sci. 2003;85:588–9.

[52] Quek S, Cook DAN, Wu Y, Marriott AE, Steven A, Johnston KL, et al. *Wolbachia* depletion blocks transmission of lymphatic filariasis by preventing chitinase-dependent parasite exsheathment. PNAS. 2022;119:e2120003119. 10.1073/pnas.2120003119). 35377795 10.1073/pnas.2120003119 PMC9169722

[53] Sucharit S, Viraboonchai S, Panavut N, Harinasuta C. Studies on the effects of tetracycline on *Brugia pahangi* in *Aedes togoi*. Southeast Asian J Trop Med Public Health. 1978;9:55–9. 705417

[54] Albers A, et al. Retarded *Onchocerca volvulus* L_1_ to L_3_ larval development in the *Simulium damnosum* vector after anti-*Wolbachia* treatment of the human host. Parasit Vectors. 2012;5:12. 22236497 10.1186/1756-3305-5-12 PMC3311148

[55] Dzimianski MT, McCall JW, Roberts R, McCall SD, Kramer LH, Genchi C, et al. Effects of ivermectin and doxycycline administered alone, together, or together plus melarsomine against adult *D. immitis* in dogs with induced infections: clinical observations. Proceedings of the American Association of Veterinary Parasitologists, 51st Annual Meeting, 15–18 July 2006, Abstract No. 66.

[56] Bazzocchi C, Mortarino M, Grandi G, Kramer LH, Genchi C, Bandi C, et al. Combined ivermectin-doxycycline treatment has microfilaricidal and adulticide activity against *D. immitis* in experimentally infected dogs. Int J Parasitol. 2008;38:1401–10. 18433753 10.1016/j.ijpara.2008.03.002

[57] Chandrashekar R, Beall MJ, Saucier J, O’Connor T, McCall JW, McCall SD. Experimental *Dirofilaria immitis* infection in dogs: effects of doxycycline and Advantage Multi^®^ administration on immature adult parasites. Vet Parasitol. 2014;206:93–8. 25218886 10.1016/j.vetpar.2014.08.011

[58] Savadelis MD, Ohmes CM, Hostetler JA, Settje TL, Zolyanas R, Dzimianski MT. Assessment of parasitological findings in heartworm-infected beagles treated with Advantage Multi R for dogs (10% imidacloprid + 2.5% moxidectin) and doxycycline. Parasit Vectors. 2017;10:245. 28526088 10.1186/s13071-017-2190-9 PMC5437498

[59] Savadelis MD, Day KM, Bradner JL, Wolstenholme AJ, Dzimianski MT, Moorhead AR. Efficacy and side effects of doxycycline versus minocycline in the three-dose melarsomine canine adulticidal heartworm treatment protocol. Parasit Vectors. 2018;11:671. 30587225 10.1186/s13071-018-3264-z PMC6307258

[60] Nelson CT, Myrick ES, Nelson TA. Clinical benefits of incorporating doxycycline into a canine heartworm treatment protocol. Parasit Vectors. 2017;10:515. 29143657 10.1186/s13071-017-2446-4 PMC5688473

[61] Nelson CT. Heartworm and related nematodes. In: Sykes JE, editor. Greene’s infectious diseases of the dog and cat. 5th ed. Elsevier; 2023. p. 1399–417.

[62] Moorehead A, Evans CC, Sakamoto K, Dzimianski MT, Mansour A, DiCosty U, et al. Effects of doxycycline dose rate and pre-adulticide, wait period on heartworm-associated pathology and adult worm mass. Par Vectors. 2023;16:251. 10.1186/s13071-023-05858-2. 10.1186/s13071-023-05858-2 PMC10369763 37491306

[63] Rawlings CA, McCall JW. Surgical transplantation of adult *Dirofilaria immitis* to study heartworm infection and disease in dogs. Am J Vet Res. 1985;46:221–4. 3970428

[64] Fricks C, McCall J, DiCosty U, Mansour A. Use of athymic nude mice in a mouse model of *Dirofilaria immitis* and its utilization in drug screening. Proceedings of the American Association of Veterinary Parasitologists, 68th Annual Meeting, Lexington, KY, 10–13 June 2023, Abstract 65.

[65] McCall JW, Malone J, Ah H-S, Thompson PE. Mongolian jirds (*Meriones unguiculatus*) infected with *Brugia pahangi*: a rich source of developing larvae, adult filariae, and microfilariae. J Parasitol. 1973;59:436. 4711663

[66] Landmann F, Voronin D, Sullivan W, Taylor MJ. Anti-filarial activity of antibiotic therapy is due to extensive apoptosis after *Wolbachia* depletion from filarial nematodes. PLoS Pathog. 2011;7:e1002351. 22072969 10.1371/journal.ppat.1002351 PMC3207916

[67] Adam R, et al. Identification of chitinase as the immunodominant filarial antigen recognized by sera of vaccinated rodents. J Biol Chem. 1996;271:1441–7. 8576136 10.1074/jbc.271.3.1441

[68] Fuhrman JA, Lane WS, Smith RF, Piessens WF, Perler FB. Transmission-blocking antibodies recognize microfilarial chitinase in Brugian lymphatic filariasis. Proc Natl Acad Sci USA. 1992;89:1548–52. 1542646 10.1073/pnas.89.5.1548 PMC48489

[69] Wu Y, Preston G, Bianco AE. Chitinase is stored and secreted from the inner body of microfilariae and has a role in exsheathment in the nematode *Brugia malayi*. Mol Biochem Parasitol. 2008;161:55–62. 18611418 10.1016/j.molbiopara.2008.06.007 PMC2577134

[70] Luck, et al. Concurrent transcriptional profiling of *Dirofilaria immitis* and its *Wolbachia* endosymbiont throughout the nematode life cycle reveals coordinated gene expression. DMC Genonics. 2014;15:1041. 10.1186/1471-2164-15-1041 PMC4289336 25433394

[71] Wu Y, Egerton G, Underwood AP, Sakuda S, Bianco AE. Expression and secretion of a larval-specific chitinase (family 18 glycosyl hydrolase) by the infective stages of the parasitic nematode *Onchocerca volvulus*. J Biol Chem. 2001;276:42557–64. 11535590 10.1074/jbc.M103479200

[72] Anderson BJ, et al. Systems analysis-based assessment of post-treatment adverse events in lymphatic filariasis. PLoS Negl Trop Dis. 2019;13:e0007697. 31557154 10.1371/journal.pntd.0007697 PMC6762072

[73] American heartworm society canine guidelines for the prevention, diagnosis, and management of heartworm (*Dirofilaria immitis*) infection in dogs. Wilmington, DE, 2024. http://www.heartwormsociety.org/veterinary-resources/canine-guidelines.html. Accession date: 17 Dec 2025.

